# “They’re thinking, well it’s not as bad, I probably won’t get addicted to that. But it’s still got the nicotine in it, so…”: Maturity, Control, and Socializing: Negotiating Identities in Relation to Smoking and Vaping—A Qualitative Study of Young Adults in Scotland

**DOI:** 10.1093/ntr/ntx245

**Published:** 2017-11-04

**Authors:** Mark Lucherini, Catriona Rooke, Amanda Amos

**Affiliations:** 1 Research Fellow, UKCTAS, Usher Institute of Population Health Sciences and Informatics, University of Edinburgh, Medical School, Teviot Place, Edinburgh, United Kingdom; 2 Visiting Fellow, UKCTAS, Usher Institute of Population Health Sciences and Informatics, University of Edinburgh, Medical School, Teviot Place, Edinburgh, United Kingdom; 3 Professor of Health Promotion, UKCTAS, Usher Institute of Population Health Sciences and Informatics, University of Edinburgh, Medical School, Teviot Place, Edinburgh, United Kingdom

## Abstract

**Objective:**

To explore the understandings of and engagement with e-cigarettes, of young adults from disadvantaged backgrounds, and how these may have an impact on existing smoking identities.

**Methods:**

Twenty-two small group and 11 individual qualitative interviews were conducted in Central Scotland with 72 16–24 year olds between September 2015 and April 2016. Participants were mostly smokers and ex-smokers from socioeconomically disadvantaged backgrounds.

**Results:**

Although most participants had tried e-cigarettes, they generally held ambivalent views about e-cigarettes and vaping. Two overarching themes were identified which helped in understanding this. Firstly, e-cigarettes were understood by the participants in relation to their existing smoking identities. Vaping was viewed as less controllable and more addictive than smoking, which did not fit with their self-identity as controlled smokers. Secondly, they felt that vaping could not replace the social and cultural importance that smoking had in their lives.

**Conclusion:**

This study suggests that though young adults from disadvantaged areas are trying e-cigarettes for various reasons, vaping is rarely sustained. Through their own experiences of vaping and their observations of others vaping, the participants perceive the behavior as endangering an existing acceptable and controlled smoking identity. Additionally, e-cigarettes were considered to be a jarring presence in existing social situations where smoking was valued. This study, therefore, provides insights into how young adults may be rationalizing their continued smoking in the face of potentially less harmful alternatives.

**Implications:**

As new and novel nicotine delivery devices, and due to their similarity to smoking, e-cigarettes have the potential to help smokers in their quit attempts. However, the findings from this study raise questions about whether e-cigarettes are regarded as having this potential by young adult smokers from disadvantaged socioeconomic environments where smoking is more commonplace and acceptable.

## Introduction

In recent years, there have been significant declines in youth smoking prevalence in many high-income countries;^1^ however, there has been relatively little decline in smoking among British young adults (16–24 year olds).^2,3^ Young adulthood can be a time of freedom to explore different identities and behaviors before the greater stability of roles and responsibilities in later adult life.^4,5^ Young adults often move in and out of smoking,^6^ and health behaviors can be abandoned or consolidated during this life stage.^7^ Several qualitative studies have found that identity construction and social factors are important in understanding young adults’ smoking,^8–11^ especially as this is a time of social and occupational transition.^12^ Young adult smokers often associate smoking with socializing and drinking alcohol, as they typically have fewer responsibilities than older adults and spend more time socializing with friends in bars, clubs, and at parties.^12,13^

These qualitative studies were undertaken before the emergence of e-cigarettes. Recent qualitative studies have focused on how these devices may be affecting smoking behaviors and identities among young adults,^14–18^ but little consideration has been given to how this intersects with circumstances of disadvantage. Recent data from England indicates that though rates of trying e-cigarettes are similar across age groups, 16–24 years olds have the lowest use of nicotine replacement therapies (NRTs).^19^ This suggests that there is less interest in quitting smoking among this age group, which is consistent with findings from Scottish national surveys^20^ and qualitative evidence.^21^ However, it also shows that young adults are trying e-cigarettes as much as older age groups who have more interest in quitting. Using e-cigarettes is an increasingly popular method to help in quitting smoking in England,^19,22^ but recent research suggests that e-cigarettes are being used by young adults for reasons other than cessation. These reasons include circumventing smoking bans,^23,24^ as fashion accessories,^25^ attractive flavors, and perceptions of vaping as “cool.”^15–18^

Smoking is often clustered into “islands” of marginalized populations where the behavior is considered almost inevitable.^26^ Indeed, in Scotland in 2015, adults living in the most deprived areas had over three times the smoking prevalence of those living in the most affluent areas (35% vs. 11%).^20^ With the relatively high prevalence of smoking in deprived areas and public health campaigns promoting smoking cessation and protecting others from second-hand smoke, smoking has become associated with class-based stigma.^27^ This stigma can be internalized by disadvantaged young smokers who are aware of how their smoking and class status interplay when they are perceived and/or judged by others.^28,29^ This leads some smokers to rationalize their smoking: for example emphasizing their “responsible” approach to smoking, such as keeping the behavior out of the sight of young children.^26^ This paper, therefore, considers how e-cigarettes are affecting the smoking identities of young adults, especially among those from disadvantaged socioeconomic backgrounds, who are more likely to be smokers.

Although the debate around e-cigarettes and their potential for smoking cessation is ongoing, in the United Kingdom a consensus seems to be emerging that e-cigarettes could provide a safer alternative to smoking.^30,31^ Current policy and practice directions in the United Kingdom are leaning towards “e-cigarette friendly” stop-smoking services and the devices are seen as important to harm reduction strategies.^32–34^ Opinions diverge in the United States, however, with the US Surgeon General’s recent report recommending that young adults be discouraged from any type of nicotine use.^35^ Recent qualitative research with British smokers reflects the uncertainty of policy-makers and practitioners about e-cigarettes’ potential role in quitting smoking. Concerns include replacing a smoking addiction with a vaping addiction^36^ or reverting to cigarettes because of the similarities between smoking and vaping.^21^ This paper builds on these recent qualitative studies by exploring the perceptions of smoking and vaping among disadvantaged young adults.

## Methods

Twenty-two friendship group interviews and 11 individual interviews were conducted with 72 young adults aged 16–25 years. Friendship groups, where participants are asked to invite one or two friends to take part in the interview, have proven effective in previous studies as they can create a more naturalistic setting for young participants when talking about tobacco issues; for example, challenging and/or supporting each other’s accounts.^37^ Most participants chose to be interviewed in friendship pairs (*n* = 16), triads (*n* = 33), or groups of four (*n* = 12). Eleven participants chose to be interviewed alone. Participants were purposively selected to represent a range of ages, employment status (students, un/employed, in training), and gender. Smokers and ex-smokers were included in order to explore how e-cigarettes were being considered in relation to cigarettes. Ethical approval was obtained from the Ethics Committee at the School for Health in Social Science at the University of Edinburgh.

Participants were not questioned directly about their socioeconomic status in order to avoid offense or embarrassment, over the disclosure of such information, which could have interrupted establishing rapport between the researcher and participant. However, to ensure that the sample came from predominantly disadvantaged backgrounds, participants were recruited mostly from community organizations in Central Scotland, which assist disadvantaged young people through skills and employability training. Interviews were arranged with the help of gatekeepers at these organizations. To obtain a spread of participants including those in work and education, other organizations including workplaces and educational institutions were contacted. Adverts were also placed on the skills exchange website Gumtree to increase recruitment of college students and those in employment. Potential participants who contacted the author conducting the fieldwork [ML] were not invited to take part, if they indicated that they came from more affluent backgrounds, identified, for example, by type of employment or educational institution. Although these markers are not always indicators of disadvantage, this screening of participant details ensured that those from more affluent backgrounds were not recruited. Recruitment material explained that the study was exploring young adults’ views about e-cigarettes, and participants were offered a £15 gift voucher. Potential participants were provided with information and consent materials and offered the opportunity to ask questions; consent forms were signed. Interviews took place mostly in community venues between September 2015 and April 2016.

The semistructured topic guide was used flexibly and explored what participants thought of e-cigarettes in relation to smoking. Interviews began with general questions about the participants’ lives and experiences of smoking and vaping. Further questions covered where and who the participants had seen vaping and/or whether and where they themselves had vaped. Questions about what participants had seen, in terms of advertising and marketing, were also covered. Accompanying the questions, the interviewer used props of four different types of e-cigarette, representing a range of disposable “cigalikes,” re-usable cigalikes (both resembling conventional cigarettes); vaporizers, which resemble marker pens; and small-box shaped devices which can be personalized and modified (commonly referred to as “box mods” by participants), to stimulate discussion. Participants used multiple terms for e-cigarettes interchangeably. In the quotes employed in this paper, “e-cigarette” generally referred to cigalikes, whereas “vaporizer” and “box mods” referred to their aforementioned descriptions. Pictures of e-cigarette advertisements and of e-cigarettes being vaped in various places were also shown to participants. These were originally included to help trigger thoughts and discussion, but this proved not to be their actual function in the interviews as most participants had seen similar devices and scenarios in their everyday life. However, the props and pictures did facilitate more natural interactions, as participants relaxed into informal conversational exchange when handling devices and leafing through pictures.

The interviews were recorded, transcribed to verbatim, and imported into Nvivo V.10 to facilitate analysis. Three rounds of coding were undertaken primarily by ML but informed at crucial junctures by all authors. Initially, and informed by a grounded theory approach,^38^ an open coding of the data was conducted, to identify recurring themes across the interviews. A sample of transcripts was similarly independently coded by another author [AA], and there was broad agreement on the identified codes. Secondly, thematic coding^39^ was employed, informed by existing literature about young adults and smoking identities. At regular meetings, the authors discussed and refined the identified codes. The thematic coding allowed the initial open-codes to be grouped into clearer overarching codes. Thirdly, focused coding^38^ enabled a nuanced identification of codes pertaining to the overarching themes. The initial coding was undertaken as data were being collected and data saturation was reached, when no new themes were identified from the initial open coding of later interviews. All participants have pseudonyms, with their smoking status, vaping status, and age given in brackets.

## Results

### Participants

Nearly half (31) of the participants were not in education, employment, or training (NEET) (Table 1). Those in employment were primarily employed in secretarial, retail, or service jobs and those in education attended colleges or further education institutions, as opposed to the major universities of central Scotland. Participants’ smoking and vaping status were assessed through the interview transcripts (Table 2). Those categorized as “ex” indicated that they previously engaged in the behavior regularly but no longer did, whereas those categorized as “ever” indicated having tried the behavior but had never become regular users. The unclear smoking and/or vaping status of some participants is due to the interviewer having limited control over who participants invited to take part and some friendship group participants being less forthcoming about their experiences than others.

**Table 1.** Participants’ Employment Characteristics

	NEET	Education/ training	Working/ volunteering	Total
Female	19	11	9	39
Male	12	8	13	33
Average age (years)	19	19.6	21	19.6
Total	31	19	22	72

**Table 2.** Participants’ Smoking and Vaping Characteristics

	Smoker	Ex-regular smoker	Ever smoked	Never smoked	Undetermined
Number of participants	44	13	9	5	1
	Vaper	Ex-regular vaper	Ever vaped	Never vaped	Undetermined
Number of participants	14	5	41	10	2

Smoking was an almost ubiquitous behavior in the lives of all participants. Some of the smokers noted very young ages at which they began (8 or 9 years old) and others noted being introduced to cigarettes by parents, grandparents, other family members, and peers. This paper considers the accounts of smoking and nonsmoking participants together as the few nonsmoking participants described their difficulties in refraining from smoking in the face of smoking family and friends, and reflected on the ubiquity of smoking in their everyday environment. As such, there was little difference between the opinions of the smokers and nonsmokers towards smoking and vaping. The following two main result sections present two of the overarching themes identified in the first and second stage of the coding process: the participants’ situating of their smoking identity in the light of e-cigarettes’ emergence and their appreciation of the social value of smoking compared to vaping. Each of these themes is unpacked by subthemes, identified in the third stage of focused coding.

### Situating smoking identity in relation to e-cigarettes

#### Between maturity and immaturity

Although smokers recognized the potential of e-cigarettes compared to existing NRT methods for quitting, many situated themselves in a transitional phase between adolescence and adulthood, where they felt that e-cigarettes were not for them. Rather they were regarded as being for older people (over 24 years) who wanted to quit, and younger people (under 16 years) prompted into trying e-cigarettes by flavors, marketing, and peer pressure. An exchange between Malcolm and Laura (both smokers, ever vaped, 17 years) demonstrated this attitude:

Malcolm: Maybe, like, older people that smoke. They probably have more patience to smoke one of them [an e-cigarette]. But I dinnae [do not] think younger people … very many of them [will use e-cigarettes].

Laura: You get all the wee [young people] jumping about, reckon they’re cool, they’re an ex-smoker and all that.

Malcolm: Like, young ones running about playing with them.

Other participants felt vaping to be initiating a new addiction and some, like Heather (smoker, ever vaped, 21 years), expressed a sense of maturity having already experienced becoming addicted to smoking: “I don’t think kids realise the effect. They must think: well it’s not a real cigarette, so it’s not that bad … I probably won’t get addicted to that. But it’s still got the nicotine in it, so...” Most participants continued to smoke, however, and Andrew (smoker, ever vaped, 21 years), using a common slang Scottish term for a cigarette—a fag—demonstrated how, despite this sense of maturity, many participants felt that they were not ready to adopt an even more responsible and mature approach by quitting smoking altogether:

A lot of people when they get to a certain age are more scared; they want to look after their body … I don’t know if it’s just a thing with me or young people …some people are just arrogant. … But, truly the only people that I’ve heard that have benefitted from these [e-cigarettes] are people that are smoking, like, 60/70 fags a day.

#### Rationalizing smoking in the face of vaping

Smokers often rationalized their smoking identities in the interviews. Alice (smoker, ever vaped, 17 years), for example, felt that she had control over how many cigarettes she consumed and, thereby, resisted an identity of being “addicted”: “I’m not addicted to cigarettes. I can smoke for, say, like a year, like consistently, every day, have a fag … I don’t get addicted.” Although smoking was considered controllable, vaping was less so, as Kevin (smoker, vaper, 21 years) and William (smoker, vaper, 25 years) discussed:

Kevin: Like you’re constantly doing it [vaping] because you can smoke it indoors.

William: And there isn’t an ending point, the only ending point is when the liquid runs out.

Kevin: Just keep puffing it …

William: It’s not like, that’s … five minutes and put it down … it still goes if it’s got a charge … I was hazed [affected by vaping] in work all the time which would never happen with a cigarette … I think that’s why I’ve kinda fallen back into smoking a bit more now cause I did have like a situation where I was like I’ve totally just [vaping too much].

Feeling in control of their smoking led participants to present their smoking as acceptable and not that “bad,” especially when other aspects of their health were positive, as Adam (smoker, vaper, 18 years) explained, “I think moderation is a good way to say how I smoke. Like I don’t power through my fags or anything I will be conservative … I think if you smoke in moderation and keep somewhat fit you shouldn’t do too bad.” In contrast to this view on smoking, many participants had observed apparently constant use of e-cigarettes amongst others. For example, Ruth (smoker, ever vaped, 23 years) said:

A lot of people I’ve seen using these just sort of seem to have them hanging out their mouth the whole time … It’s so easy to pick these up … you don’t even need to go outside … I think I’d probably be … smoking more than I usually do but just through not noticing, not actively trying to smoke more.

Interestingly, Ruth did not differentiate “smoking” and “vaping” in the above quote. This was similar for many of the participants and demonstrates their attitude towards vaping as doing little to change existing smoking behaviors and identities. However, a few participants had found controlling vaping to be straightforward. Although Ellen (smoker, ever vaped, 22 years) also saw a seemingly increased addiction in other e-cigarette users, she was bemused at this behavior as she was able to equate vaping duration with a cigarette: “You kinda reach a natural end of … ‘right, I’ve been using this for four minutes, that’s like a fag, I’m going to put this away now’, so it was weird to see someone just sitting constantly [vaping].”

#### Quitting smoking and quitting vaping

Many participants felt that they would be able to quit smoking, given their “acceptable” level of smoking and relative young age. The ability to quit vaping was seen as less certain. Participants such as Jane (smoker, ever vaped, 19 years) were often puzzled as to why people appeared not to cut down vaping and try to stop altogether: “anybody that I’ve heard has stopped smoking with them they’ve been on them since they stopped smoking for like however many years, they’ve been on them since … they’ve just constantly stuck to that.” Likewise, David (smoker, ever vaped, 19 years) described losing his confidence to quit using an e-cigarette after observing the seeming failure to do so among friends:

I was going to try completely stop smoking normal cigarettes and then after a while come off the e-cigarette but I know plenty people that have been on the e-cigarette for over two years now, so they’re just as addicted to that as they are normal cigarettes.

Contrary views to the addictiveness of vaping were present among a few participants. For example, Steven (ex-smoker, vaper, 20 years) described successfully transitioning from smoking to vaping using a box mod e-cigarette, and his ongoing process of lowering the nicotine content of his e-liquids to eventually reach zero nicotine and stop vaping. Steven was one of four participants to report having quit smoking with the use of an e-cigarette and, along with the other three, reported a specific personal motivation for quitting. For Steven, this was a sudden realization of a drop in fitness. For the others, it involved reaching a milestone age, impending parenthood, and a diagnosis with Type 1 diabetes. Three of these four participants used a box mod e-cigarette. However, few others noted having tried a box mod, and so their experiences of e-cigarettes mostly lay with cigalikes and vaporizers. The high up-front cost and what many perceived as extravagant features, compared to the more affordable and accessible e-cigarette models, were reasons given for avoiding box mods. Conversely, the few users of box mods referred positively to the long-term cost-effectiveness, sophisticated features, and personalization of the devices, and believed them to be the most effective for smoking cessation.

### The social value of smoking

#### Alcohol and socializing

Smoking while drinking alcohol was an almost unquestioned necessity among the participants, and quit attempts using e-cigarettes often collapsed when drinking:

Graham (smoker, ex-vaper, 19 years): I tried to stop [smoking] with a vapouriser but it only went so far … It doesn’t work when you go out drinking.

Gregory (smoker, ex-vaper, 21 years): It really doesn’t … Used a vapouriser to try and stop. It wasn’t good enough. Like Graham said, when you’re going out for a drink it just doesn’t do it.

Participants also mentioned e-cigarettes being left behind when preparing for a night out or being neglected while out. Indeed, smoking was an important social interaction for participants and some lamented the more individualized behavior of using e-cigarettes:

Julia (smoker, ever vaped, 21 years): Well it was a big social thing, when you had a fag, and you went out for a drink and a quick fag.

Kate (ex-smoker, ever vaped, 19 years): You’d be like, ‘excuse me, have you got a light’? And now, it’s like, you just keep [to] yourself, you don’t see people speaking.

Interviewer: And do you think the e-cigarettes and the vapourisers have contributed to that?

Kate: Probably.

Julia: Aye.

Kate: It must’ve, ‘cause you can’t go to someone, ‘can I charge one?’! … ‘Have you got a spare battery’? You know what I mean, it’s gone really weird.

Though sharing cigarettes and lighters could start conversations, sharing e-cigarettes was perceived to be a conversation ender. A few participants did note positive aspects of the individuality of e-cigarettes. For example, Daniel (ex-smoker, ever vaped, 19 years) felt that personalizing devices meant they were less likely to be shared and so it was easier to avoid social smoking cues as he maintained his attempt at quitting smoking. Some participants had integrated vaping more successfully into their lives. William and Kevin had become friends through a common enthusiasm for vaping. However, both still smoked and were not intending to quit in the near future. Several others mentioned the likelihood of “going back to fags,” at times of drinking and stress, should they try to quit using e-cigarettes.

#### Stress and smoking

Just as cigarettes were synonymous with drinking alcohol, they were also often understood as important stress relievers for the participants. Many described the everyday stress of their lives, usually due to their precarious working and living circumstances. For instance, one group mentioned that just living in a deprived area made them want to smoke, whereas others noted that smoking, along with other substance use, was just part of what they encountered in their daily lives. Fred (smoker, vaper, 24 years) provided a specific insight into this stress when he talked about being nervous before a job interview: “so I’ll have a fag, and stand outside for ten minutes … but I try and not use the e-cig ‘cause it doesn’t help with the nerves … A fag kind of helps you a wee [small] bit there.” Fred did not like smoking before an interview, aware that the smell of smoke on his body and clothes served to stigmatize him to interviewers, however, he felt unable to escape this felt stigma, as for him there was no effective replacement for a cigarette.

Reinforcing the idea of smoking as more controllable than vaping, participants often explained their smoking in terms of taking a break to relieve stress. Smoking a cigarette was often described as merely something to do while having their break—as opposed to being driven by nicotine addiction. Jane (smoker, ever vaped, 19 years) and Karen (smoker, ever vaped, 20 years) worked in the same office. They described taking three or four smoking breaks a day. They compared their break schedule with the much more frequent vaping breaks of a colleague who they felt was more addicted to vaping than he had been to smoking, and so, to them, the frequency of his vaping breaks seemingly diminished their value. A 5-min break outside with a cigarette therefore retained value for Jane and Karen, as the prospect of switching to e-cigarettes meant that control over their addiction would be lost.

## Discussion

A key finding of this study is that e-cigarettes and vaping did not fit into the lives of most of the participants as possible smoking cessation aids or as replacements for cigarettes and smoking. Smoking had come to be acceptable and manageable in many participants’ lives and identities. E-cigarettes were perceived as endangering this, threatening to lull them into an identity of less control characterized by an even greater addiction than that of smoking. Participants, therefore, stressed their self-identity as “controlled smokers” rather than “out-of-control vapers.” Avoiding e-cigarettes could be a marker of maturity among these participants, relative to the immaturity of younger people who they felt were unable to make informed decisions about potentially addictive behaviors. This finding is contrary to some other qualitative studies on young adults and e-cigarettes which find that the ability to perform “smoke tricks” and other markers of “vaping trendiness” were important factors for use.^17^ Nonetheless, the participants also distanced themselves from even more mature, older people, who might use e-cigarettes as cessation aids. This form of identity work builds on the findings of Thomson et al.^26^ that in response to perceived moral imperatives to demonstrate some compliance with smoking disapproval, smokers attempt to craft identities as “responsible” or “considerate.”^40^ The participants in this study stressed their continued smoking as a more responsible option than transitioning to vaping. This raises the possibility that negative attitudes towards e-cigarettes and vaping may be entrenching existing justifications for continued smoking among young adults from disadvantaged areas.

Nonetheless, e-cigarettes were used by some participants in attempts to quit smoking. These attempts were generally short-lived as e-cigarettes and vaping failed to adequately replace the important social aspects of smoking, which remained an important social “lubricant.”^12^ In contrast, the use of e-cigarettes in social settings was perceived to impede social interaction. Stress was normal for these young adults, as they attempted to secure employment and establish more stable routines. As has been found in previous studies of disadvantaged smokers,^41,42^ smoking was experienced and valued as a stress reliever. In contrast, participants found that vaping rarely offered an effective alternative. Other research has indicated that young adult smokers believed e-cigarettes to be effective replacements for cigarettes;^18^ however, the disadvantaged circumstances of the participants in this study, seemingly increased the “resilience” of smoking,^43^ so that vaping was unsustainable. Some participants formed social practices around a shared enthusiasm for vaping. However, several of these participants were dual users of cigarettes and e-cigarettes, and recent research has suggested that dual use may be a lasting behavior in itself rather than a stage of quitting smoking.^44^ Type of e-cigarette may also have affected the participants’ views. The more expensive and sophisticated box mods were financially inaccessible to many, but users did note their greater potential as smoking cessation aids. Future research should consider examining the different models of e-cigarettes and their uses, independently, rather than treating e-cigarettes as homogenous devices.^45^

The evidence base around e-cigarettes is constantly developing and so these findings are limited to a specific geographical context and the time when they were gathered and analyzed. These qualitative data from a small purposive sample cannot be used to generalize to the Scottish or UK population. Access to and attitudes towards e-cigarettes and uptake of vaping may differ by socioeconomic status and so have an impact on health inequalities.^46,47^ The participants’ attitude to e-cigarettes and vaping was influenced by their living in disadvantaged areas of Central Scotland, where smoking is more of a social norm. More research is needed to explore the variable meanings of e-cigarettes and vaping by socioeconomic status.

These findings are particularly pertinent to understand how e-cigarettes are being understood among a population with particular attitudes of disapproval towards smoking with simultaneously high rates of smoking. The fact that participants felt switching to e-cigarettes did little to alleviate an identity of being “addicted” confounds recent commentaries that e-cigarettes offer a route to denormalize smoking without stigmatization.^48^ Although there is yet no clear consensus on the health equity impact of e-cigarettes,^46^ there remain fears that e-cigarettes will be less accessible to those on lower incomes and exacerbate existing health inequities related to smoking.^49^ Additionally, the fact that the participants’ attitudes towards e-cigarettes were being informed by their perception of addiction is supported by evidence from another qualitative study^15^ that finds young adults used their own embodied experiences of vaping to inform their opinions. Prevailing public health views that e-cigarettes are likely to be less harmful than combustible tobacco products^26–28^ are, perhaps, not well disseminated among young adults and this lack of information may well be exacerbated by circumstances of disadvantage. Indeed, many of the participants noted limited access to technology and social media. The findings in this study highlight the importance of exploring the social context of disadvantaged young smokers, as the health inequity impact of e-cigarettes cannot be reduced to only matters of cost, pricing, and access. The participants’ decisions to reject e-cigarettes were based on ideas of addiction, identity, and stigma, along with existing social relations in which cigarettes and smoking were necessary and irreplaceable. Therefore, e-cigarettes should not distract from continued efforts to tackle the underlying social and cultural reasons for smoking among disadvantaged young adults.

## Funding

This work was supported by a Cancer Research UK (CRUK) Tobacco Advisory Grant (TAG) award. The reference for the award is C3721/A19466.

## Declaration of Interests

*None declared*.
